# Optimizing SNR for multi‐metabolite hyperpolarized carbon‐13 MRI using a hybrid flip‐angle scheme

**DOI:** 10.1002/mrm.28194

**Published:** 2020-02-03

**Authors:** Lauren M. Smith, Trevor P. Wade, Lanette J. Friesen‐Waldner, Charles A. McKenzie

**Affiliations:** ^1^ Department of Medical Biophysics University of Western Ontario London Ontario Canada; ^2^ Division of Maternal Fetal and Newborn Health Children’s Health Research Institute London Ontario Canada; ^3^ Robarts Research Institute University of Western Ontario London Ontario Canada

**Keywords:** carbon‐13, hyperpolarized MRI, metabolism, pyruvate, RF pulse design

## Abstract

**Purpose:**

To improve the SNR of hyperpolarized carbon‐13 MRI of [1‐^13^C]pyruvate using a multispectral variable flip angle (msVFA) scheme in which the spectral profile and flip angle vary dynamically with time.

**Methods:**

Each image acquisition in a time‐resolved imaging experiment used a unique spectrally varying RF pulse shape for msVFA. Therefore, the flip angle for every acquisition was optimized for pyruvate and each of its metabolites to yield the highest SNR across the acquisition. Multispectral VFA was compared with a spectrally varying constant flip‐angle excitation model through simulations and in vivo. A modified broadband chemical shift‐encoded gradient‐echo sequence was used for in vivo experiments on six pregnant guinea pigs. Regions of interest placed in the placentae, maternal liver, and maternal kidneys were used as areas for SNR measurement.

**Results:**

In vivo experiments showed significant increases in SNR for msVFA relative to constant flip angle of up to 250% for multiple metabolites.

**Conclusion:**

Hyperpolarized carbon‐13 imaging with msVFA excitation produces improved SNR for all metabolites in organs of interest.

## 1 INTRODUCTION

The placenta produces a variety of biomolecules during pregnancy that are important for both maternal and fetal metabolism.^1^ In vivo assessment of placental metabolism would allow us to characterize healthy metabolism at all stages of pregnancy and investigate potential placental metabolic abnormalities in conditions such as intra‐uterine growth restriction.^2^ Imaging assessment of the placenta during pregnancy is generally limited to the nonionizing imaging modalities of ultrasound and MRI. These imaging techniques can contribute structural information and limited functional data about the placenta.^2^, ^3^

Hyperpolarized carbon‐13 MRI (HP^13^C‐MRI) is an ideal imaging modality for real‐time in vivo monitoring of metabolism. Using dissolution dynamic nuclear polarization methods, it is possible to temporarily increase the signal up to 10 000 fold and acquire images of the distribution of molecules enriched with the hyperpolarized substrate.^4^ This technique has a variety of applications in research, including tumor monitoring, inflammation, cardiac disease, and fetoplacental development.^5^, ^6^

One of the more common HP^13^C‐MRI substrates is [1‐^13^C]pyruvate, which, following an intravenous injection, allows us to resolve signal from its metabolites: lactate, alanine, and bicarbonate. Pyruvate metabolism is a key step in glycolysis and is indicative of normal and abnormal cell function. The metabolic by‐products of pyruvate follow different metabolic pathways, some aerobic and some anaerobic, and the metabolic fates of these biomolecules provide insight to the biological state of the region or organ of interest. The metabolic conversion rate constant from pyruvate to a metabolite (for example, K_PL_ is the conversion rate of pyruvate → lactate) can be calculated from the time‐resolved measurements of the different metabolites and provides a quantitative measure of metabolism related to enzyme concentrations in vivo.^7^ Measuring the entire concentration time curve of each metabolite is necessary for a robust sampling of data in order to calculate metabolic rates; as such, a sufficiently high SNR must be maintained throughout the entire acquisition.

The metabolites of pyruvate we wish to image exist at distinct chemical shifts (between −9.7 and 12.6 ppm relative to pyruvate). Because the metabolism of the injected pyruvate occurs rapidly, the relative abundance of each metabolite changes over the course of the experiment. The metabolites are typically found in quantities over 6‐fold lower than pyruvate. In past experiments we took advantage of this by using a spectrally varying RF pulse, such that pyruvate is exposed to a smaller flip angle than its metabolites that are lower in abundance.^5^ This flip‐angle scheme is spectrally varying but constant in time and will be referred to as the constant flip angle (CFA) scheme.

The rapid T_1_ decay of the hyperpolarized state provides challenges to maximizing SNR. Previous HP^13^C‐MRI studies^8^, ^9^, ^10^, ^11^, ^12^, ^13^, ^14^ have addressed this by proposing variable flip angle (VFA) schemes that increase the flip angle over time for one metabolite. These studies typically use a spectral spatial RF pulse shape and vary the pulse amplitude at each excitation, continuously increasing to 90° to maintain a constant signal from a single spectral peak of a hyperpolarized ^13^C molecule despite the rapid decay of polarization.

We aim to expand on the CFA scheme to optimize SNR in HP^13^C‐MRI experiments by introducing an approach that allows us to deliver optimized time‐varying flip angles to each metabolite by taking advantage of the spectral variance of CFA. This will be referred to as the multispectral variable flip angle (msVFA) scheme. The individual trajectories for each metabolite using msVFA are calculated using previously published VFA methods and are applied to each metabolite simultaneously by progressively varying both the shape and amplitude of the spectrally varying pulse. This allows us to produce a distinct VFA trajectory for each metabolite and apply the SNR benefits associated with VFA to all metabolites simultaneously.^15^

In this study we compare the msVFA to the CFA scheme. We have chosen to compare CFA to msVFA, as it allows us to compare the SNR for all four metabolites of interest simultaneously. Additionally, this comparison allows us to introduce an alternative to the spectral spatial RF method as an improvement of the current IDEAL technique. Therefore, we are interested in investigating the addition of time‐varying flip angles to the CFA method to provide optimal SNR for multiple metabolites simultaneously.

We hypothesize that the msVFA scheme will provide SNR equivalent to VFA for each metabolite; therefore, it will significantly increase SNR throughout the experiment for each metabolite compared with the CFA scheme. We believe that msVFA has the potential to improve HP^13^C‐MRI image quality in a variety of research applications, including (but not limited to) fetoplacental research.

## 2 METHODS

### 2.1 Simulations

**Figure 1:**
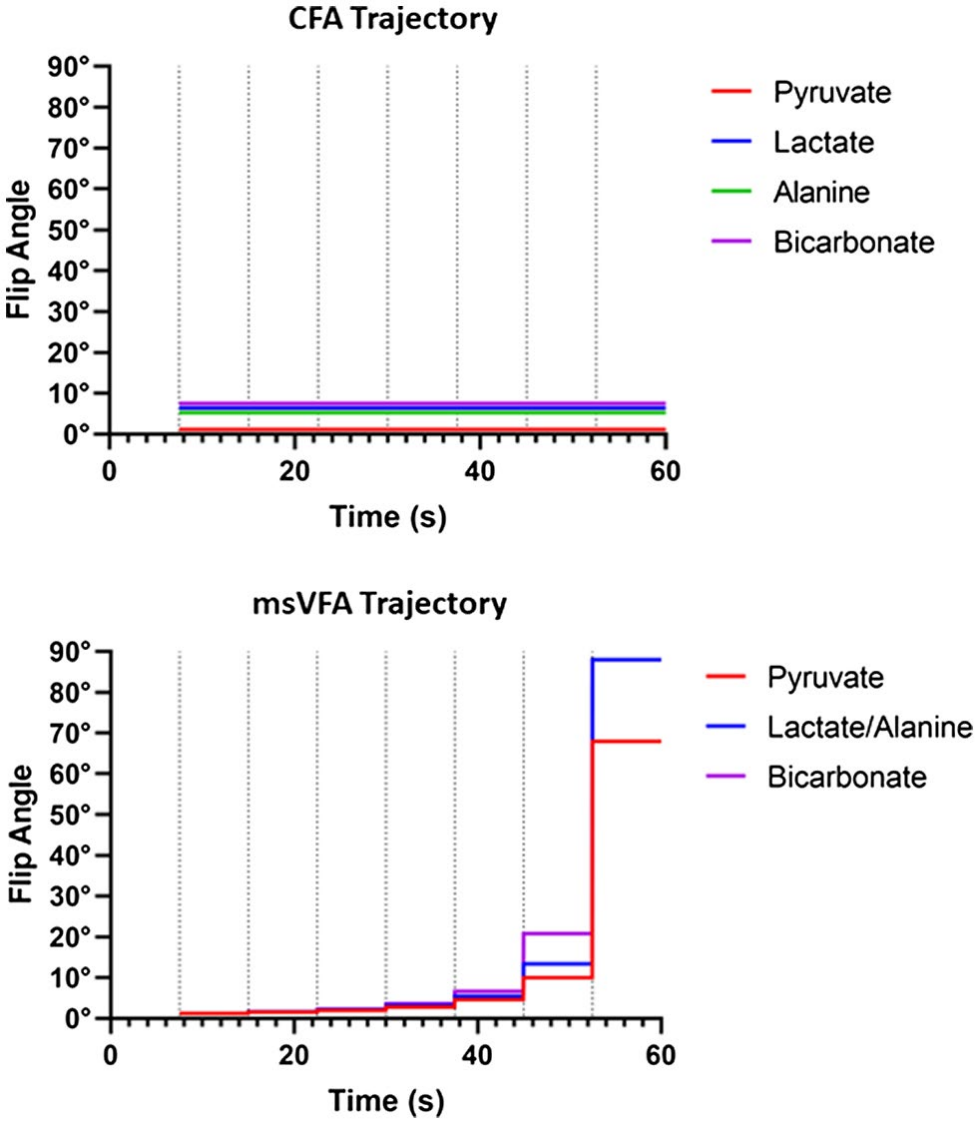
The desired flip‐angle trajectories for each metabolite are shown for constant flip angle (CFA; top) and multispectral variable flip angle (msVFA; bottom) strategies. These flip angles were achieved using a double Gaussian RF pulse. Dotted vertical lines indicate the start time of each acquisition

Flip angles for each metabolite were calculated using the method described by Xing et al,^13^ which takes into account both the T_1_ decay and metabolic conversion of each metabolite. This method was used to calculate the flip angles that would optimize SNR for each metabolite throughout the experiment. The calculated flip angles for the msVFA scheme were compared with a CFA scheme previously described by Friesen‐Waldner et al^5^ Figure 1 shows the flip‐angle trajectories applied in this work for both of these methods.

Bloch equations were used to simulate the SNR of different metabolites using MATLAB 2018a (MathWorks, Natick, MA). Equation 1 was used to estimate the SNR at each nth time point for every metabolite (met), in which the concentration of each metabolite was calculated using the estimated T_1_ decay and metabolic conversion rates found in the literature.^16^, ^17^ The predicted SNR resulting from using the implemented flip angle in msVFA was compared with the predicted SNR using the exact VFA for each metabolite.

(1) $$SNR_{\mathrm{met}}\left(n\right)=\left[met\left(n\right)\right]e^{-\frac{\left(n-1\right)TR}{T_{1met}}}\left(\sin\theta \right)\cos\;\theta^{n}$$

### 2.2 Multispectral VFA implementation and verification

In the msVFA experiments, we apply a unique RF waveform for each image acquisition.^15^ Additional improvement would likely be possible by applying a unique RF waveform for each excitation of each image. This may be feasible for single‐shot pulse‐and‐acquire‐type sequences but becomes impractical to implement for the thousands of custom RF pulse shapes required for the sequence used here.

The double Gaussian pulse allows us to implement unique flip angles for each metabolite; however, the double Gaussian pulse shape limits our accuracy in delivering the exact calculated flip angles to every metabolite. If we were only interested in observing two or three metabolites, the double Gaussian pulse could be used to deliver the exact calculated flip angles; however, with four metabolites we have to make some approximations due to the different spectral positions. This is why lactate and alanine are delivered the same flip angle in msVFA experiments.

**Figure 2:**
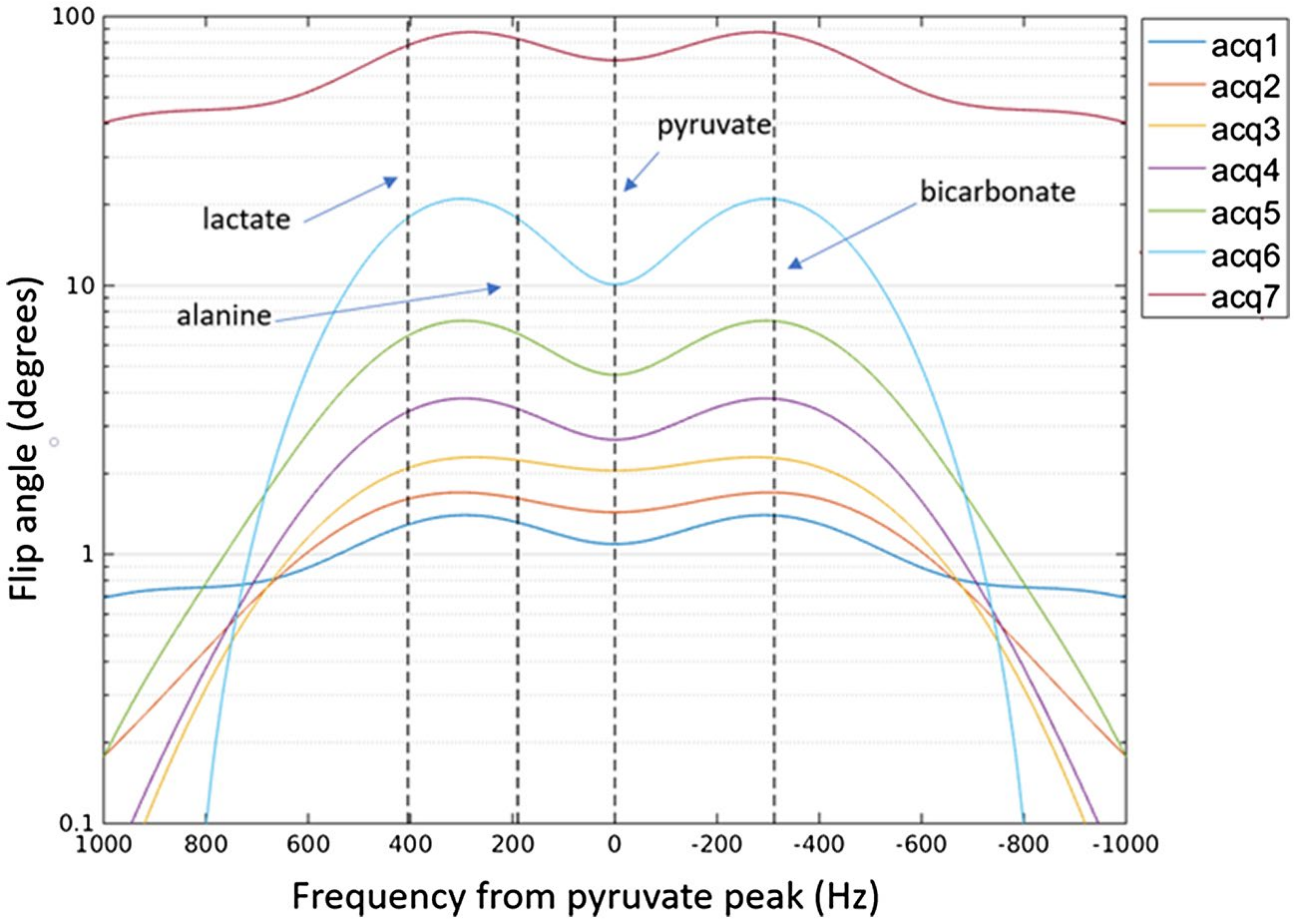
Double Gaussian RF spectral profile used in each msVFA acquisition, centered on the pyruvate peak. The resonant frequency of each metabolite is indicated by vertical dashed lines. The magnitude and shape of the RF profile progressively changes with each acquisition to follow the msVFA trajectory

For our ^13^C acquisitions, we used a quadrature volume transmit and receive coil (12 cm diameter, 19 cm long).^18^ The desired flip angles were applied to each metabolite using double Gaussian RF spectral profiles designed for each acquisition. The RF profiles for each acquisition are shown in Figure 2.

These RF pulses were implemented in a version of our previously described chemical shift–encoded [1‐^13^C]pyruvate imaging pulse sequence.^5^, ^15^, ^18^ The modified pulse sequence was tested on a phantom containing 7 mol/L thermal [1‐^13^C]sodium acetate doped with a gadolinium‐based contrast agent^19^ to ensure that RF pulses were scaling appropriately to prescribed flip angles over time. Frequency sweeps of the RF pulses were used to determine the RF amplitude at various chemical shifts relevant to [1‐^13^C]pyruvate imaging. The pulse sequence was run at a longer TR (1.5 seconds) compared with hyperpolarized experiments to permit for the T_1_ recovery of the thermal phantom.

### 2.3 Animal experiments

Six pregnant adult guinea pigs (gestational age = 48.2 ± 11.7 days, number of pups = 3.4 ± 0.9) were imaged under the approval of the institution’s Animal Care Committee. Due to a technical failure of the RF coil in one experiment, we excluded one animal from analysis, leaving a total of 5 guinea pigs and 17 placentae. Animals were anesthetized using 4.5% isoflurane with 2‐L/min O_2_ and maintained between 1.5% and 2.5% isoflurane with 2‐L/min O_2_. Animal vital signs (breathing, heart rate, temperature, and blood oxygenation) were monitored throughout the experiment. Experiments were done at approximately the same time of day, and all animals were fasted for 2 hours before the experiment to standardize their metabolic state at the start of the experiment. All animals underwent an ultrasound examination 3 days before the MRI experiment to confirm pregnancy.

Each animal received two pyruvate injections during an experiment, one imaged with CFA and the other with msVFA. The order of the msVFA and CFA acquisitions were randomized between experiments. The second injection occurred approximately 1 hour after the first, to allow time for hyperpolarization of the second dose of pyruvate.

For each injection, [1‐^13^C]pyruvic acid (Cambridge Isotope Laboratories, Tewksbury, MA) containing 15‐mM OX63 trityl radical (Oxford Instruments, Abingdon, United Kingdom) and 1‐mM ProHance (Bracco, Milan, Italy) was hyperpolarized using the Hypersense DNP polarizer (Oxford Instruments). A total of 75 mg/kg of the hyperpolarized 250‐mM [1‐^13^C]pyruvate solution was delivered as a bolus injection through intravenous catheter into the saphenous vein over approximately 12 seconds, approximately 15 seconds after the dissolution had been released from the polarizer. Imaging was initiated 7.5 seconds after the start of pyruvate injection. An image was acquired every 7.5 seconds following the first acquisition, resulting in a total of seven images acquired at different time points. Hyperpolarized imaging was done using a 3D multiphase broadband fast gradient recalled multi‐echo pulse sequence with the following parameters: FOV = 20 × 0.6 cm, slice = 8.5 mm, bandwidth = 8.93 kHz, echoes = 8, number of excitations = 1, echo train length = 4, first TE = 4.2 ms, echo spacing = 1.1 ms, and acquisition time = 7.5 seconds.

The T_1_‐weighted gradient echo (TR/ TE = 5.1 ms/2.4 ms, flip angle = 15°, number of averages = 4, slice thickness = 0.9 mm, total imaging time ~ 7 minutes) and T_2_‐weighted spin echo (TR/TE = 2000 ms/120 ms, number of averages = 2, slice thickness = 0.9 mm, total imaging time ~ 7 minutes) images with 0.875 × 0.875 mm^2^ in‐plane resolutions were obtained as anatomical references for the ^13^C images. All experiments were done on a 3T MRI system (Discovery MR750; GE Healthcare, Waukesha, WI). The ^1^H images were acquired using a 32‐element human cardiac coil array (In Vivo, Gainesville, FL), and ^13^C images were acquired using a custom‐built ^13^C birdcage coil (Morris Instruments, Ottawa, Canada). Polarization levels were measured with a multiple‐quantum coherences spectrometer shortly after the dispensation of the sample for each injection.

### 2.4 Signal‐to‐noise ratios

The SNR of the in vivo images was calculated as the mean signal in a region of interest (ROI) divided by the SD of signal in a signal‐free ROI placed outside the animal. The ROIs were drawn on the T_1_ images for each placenta, maternal liver, and maternal kidney, and then transferred to the HP^13^C images. The SNR was calculated for each metabolite and acquisition. Paired *t*‐tests were done to compare the SNR for each metabolite in each ROI. Significance was defined at 0.05 for this test.

## 3 RESULTS

Using the msVFA double Gaussian pulse, we are able to achieve flip angles very similar to those of the optimal VFA calculated for each metabolite. Bloch simulations found that the predicted SNR using the flip angles implemented with msVFA was very similar to the predicted SNR using the exact VFA calculated for each metabolite. The SNR ratio summed over time for each metabolite using msVFA compared with the true VFA are as follows: 97% for pyruvate, 89% for lactate, 97% for alanine, and 95% for bicarbonate.

**Figure 3:**
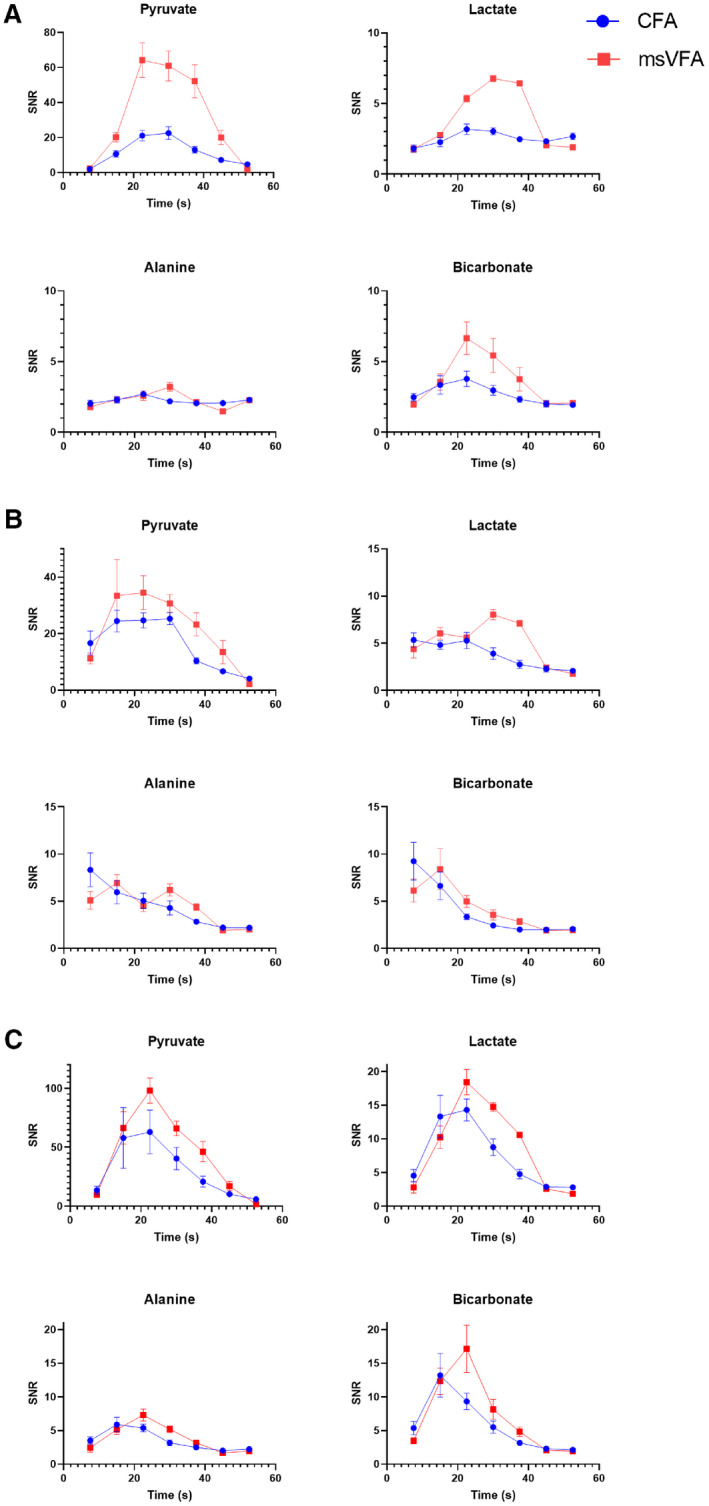
Mean SNR for each metabolite averaged over all placenta (A), maternal liver (B), and maternal kidney (C) regions of interest (ROIs). The mean SNR at each time point may be compared between msVFA (red squares) and CFA (blue circles) acquisitions. A, The increased SNR provided by the msVFA acquisition is apparent for all metabolites, except alanine. This is likely due to limited production of alanine in the placenta providing very little metabolite signal, which could be increased by the use of msVFA. B, The increased SNR provided by the msVFA acquisition is apparent for pyruvate and lactate, although there is limited improvement for alanine and bicarbonate. C, There appears to be an increased mean SNR using msVFA for all metabolites in maternal kidney ROIs; however, these trends were not found to be significant. Note that for all ROIs, the y‐axis SNR scale is larger for pyruvate due to a larger amount of signal from pyruvate compared with other metabolites

An increase in SNR in the placentae was observed for all metabolites using msVFA at time points up to 37.5 seconds, as displayed in Figure 3. We observed statistically significant (*P *< 0.00005) increases in SNR for pyruvate, lactate, and bicarbonate signal in the placentae using the msVFA acquisition compared with the CFA acquisition. An exception of this SNR increase in the placentae was the alanine signal; however, placental alanine SNR was very low, suggesting that very little alanine was being produced in the placentae, and as such it is not possible to increase the SNR when negligible signal is present.

We noticed significant increases of pyruvate and lactate SNR in the maternal liver (*P *= 0.005 and *P *= 0.006, respectively). We did not find a significant difference in the SNR of alanine and bicarbonate in the maternal liver, but low SNR indicates that very little alanine and bicarbonate were produced in the maternal liver. We observed a significant increase in pyruvate SNR using msVFA in the maternal kidneys (*P *= 0.015) and noticed trends of increased mean SNR using msVFA for all metabolites (see Figure 3).

We did not typically see an increase in SNR using msVFA at the last time point (52.5 seconds), as there was not much hyperpolarized signal left to acquire at this time point. In some cases, we saw an increased SNR in the CFA images at 52.5 seconds, and this may be due to the fact that CFA is less efficient at using all of the signal by the end of the scan, leaving “wasted” signal. The SNR was not corrected for polarization level in this analysis, because all polarization measurements were between 5% and 7%, and the polarization level was not significantly different between msVFA and CFA, as determined by a paired *t*‐test (*P *= 0.15).

**Figure 4:**
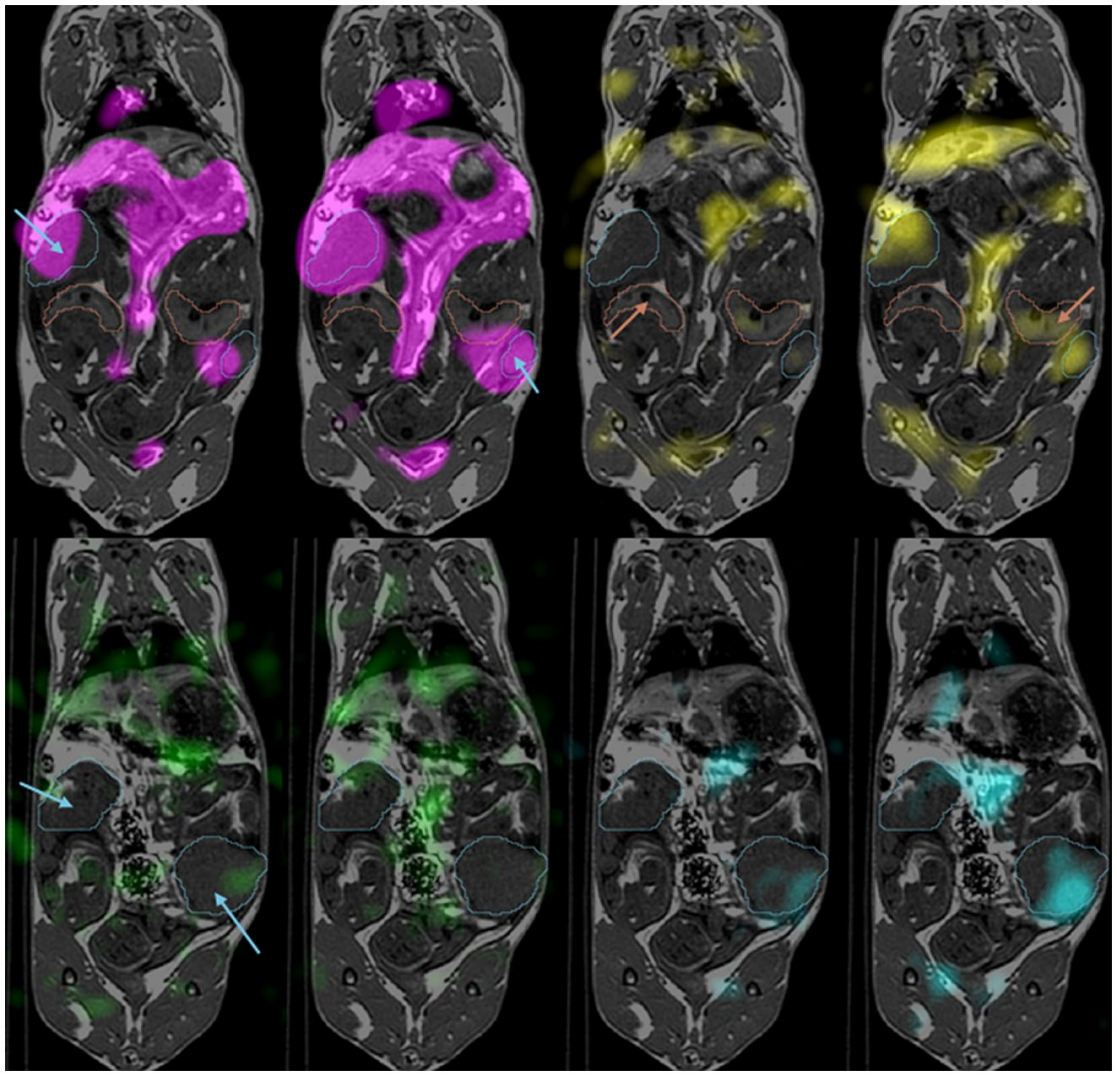
Typical hyperpolarized ^13^C metabolite images overlaid on axial T_1_ of the same animal at 30 seconds after injection. Four pairs of images are shown here for each metabolite: magenta, pyruvate (top left); yellow, lactate (top right); green, alanine (bottom left); and cyan, bicarbonate (bottom right). For each pair of images, the image on the left was acquired using CFA and the image on the right acquired using msVFA. The image pairs have identical window and level for each metabolite. Placentae are indicated by blue arrows and outline, and fetal livers are indicated by red arrows and outline. A different slice is shown for alanine and bicarbonate, to show increased bicarbonate signal in the placenta using msVFA

Qualitatively, we can appreciate that the increased SNR in images collected using the msVFA acquisition allowed us to better discern the signal in less perfused and/or metabolically active areas compared with the lower SNR images acquired using the CFA method. This allowed us to better distinguish low metabolism values from noise and to detect small changes in metabolism. We were able to distinguish signal in the fetal livers more often in images acquired using msVFA compared with CFA. An example of this is displayed in Figure 4, where we notice more signal in placentae and fetal livers in the image acquired using the msVFA technique.

## 4 DISCUSSION AND CONCLUSIONS

In this study, we demonstrated that a spectrally varying dynamic flip‐angle scheme individually optimized for each metabolite increases the SNR for all metabolites in time‐resolved metabolic imaging. We have shown up to 250% increases in SNR in pyruvate, lactate, and bicarbonate images of the placentae, and demonstrated similar SNR increases for metabolites in the maternal liver. The lack of alanine signal in the maternal liver may be related to the known decrease of alanine aminotransferase enzyme activity in the liver that occurs during pregnancy, although this has only been reported on in human studies.^20^, ^21^ The increased incidence of signal seen in fetal livers using msVFA compared with CFA leads us to believe that msVFA may be useful for metabolic analysis of fetal organs, where otherwise the signal may not be distinguishable from noise.

The msVFA approach combats the rapid decay of hyperpolarized signal while accounting for the variation in metabolite concentrations and T_1_ rates in hyperpolarized [1‐^13^C]pyruvate MRI. The delivery of flip angles optimized for different metabolites in HP^13^C‐MRI builds on studies that have shown the advantage of VFA techniques to counteract the rapid hyperpolarized decay.^8^, ^10^, ^12^, ^13^, ^22^ In these previous studies, VFA was optimized for one metabolite, usually either pyruvate or lactate, leading to suboptimal flip angles for other metabolites.

Alternative approaches to optimize SNR for multiple metabolites have used spectrally selective RF pulses, which apply a unique flip angle for each metabolite but only excite one metabolite per RF pulse.^10^, ^11^, ^13^, ^14^ These approaches to date have only been used to optimize up to two metabolites, usually pyruvate and lactate, and are usually implemented for imaging 2D slices. In our application of fetoplacental imaging, 3D imaging has the advantage of ensuring we can image multiple placentae and fetuses, which would be difficult if limited to a single 2D slice. The msVFA technique uses a spectrally varying flip angle, which delivers unique flip angles for each metabolite during every RF excitation. The msVFA method does not limit the number of metabolites for which we may use optimized flip angles, and scaling up msVFA for more metabolites would be possible with a change in the RF excitation pulse shape. The current implementation of msVFA does not use the exact VFA flip angles for each metabolite due to limitations of the double Gaussian RF pulse. Delivering the exact desired flip angles may be possible, as one could imagine using a more sophisticated RF pulse design; however, more complex pulses may be associated with additional complications such as higher specific absorption rate and likely longer pulses, which we want to avoid in hyperpolarized experiments. Although both methods of achieving multimetabolite VFA are valid, unique advantages and weaknesses exist for either method. We consider our stepwise msVFA to be a step toward a fully optimized VFA for 3D volumes.

The msVFA technique may be generalized for any set of metabolites in hyperpolarized imaging, assuming that a rough estimate of the T_1_ and metabolic rate constant is known. This would be valuable for hyperpolarized imaging of any metabolite, particularly if the end goal is quantitative analysis. The in vivo T_1_ rates of these metabolites are difficult to measure and not well characterized; however, the msVFA optimization is dependent on the ratio of TR/T_1_, and hyperpolarized imaging sequences typically use TR << T_1_ due to the limited lifetime of hyperpolarized signal. Therefore, the msVFA optimization calculation is relatively insensitive to errors of in vivo T_1_ estimation. Similarly, flip‐angle optimization calculations from Xing et al^13^ used the assumed metabolic rate constant values in the estimation of the effective T_1_, which is inversely related to the rate constant. These metabolic rate constants are not well known in vivo, but as previously, we do not expect errors in rate constant estimation to affect SNR, as TR << T_1eff_ in our sequence.

The metabolic rate constants measured in vivo are typically on the order of 10^−2^ or 10^−3^ s^−1^, and precision is important when quantifying such small values. We predict that the increased SNR produced in images acquired with msVFA will improve the precision of metabolic rate fitting, enhancing the ability to detect small changes in these values. A future application of this work would be to quantify and compare metabolic rate constant values in diseased and healthy placentae using the msVFA acquisition.

In this study we have a large degree of biological variation among our animals, including different maternal ages, gestational ages, and number of fetoplacental units. We chose to include a diverse population for in vivo experiments to demonstrate that the advantages of using msVFA are applicable to different populations, meaning that the msVFA would not bias the results when comparing two groups of animals.

For these ^13^C acquisitions, we were able to use the birdcage coil to limit the guinea pig volume we wished to image. If we were to translate this technique to application in large animal or human studies, the RF pulse would need to be redesigned to include spatial selectivity. This adaptation is an important topic for future work.

In summary, we have demonstrated that msVFA results in improved overall SNR for all metabolites in in vivo hyperpolarized [1‐^13^C]pyruvate MRI. This was achieved using a spectrally varying RF pulse that increases in amplitude over the duration of the scanning protocol, preserving hyperpolarized ^13^C signal more effectively than a flip‐angle scheme that is constant over time. Although we have focused on reporting the SNR for ROIs in the placentae, maternal liver, and maternal kidneys, this technique could improve SNR for any organ where metabolism is occurring. Additionally, this technique may be generalized for metabolic imaging of any hyperpolarized substrate. The msVFA technique improves hyperpolarized ^13^C images to allow better quantitative analysis of metabolic rates, and this is an important step in translation of HP^13^C‐MRI to clinical applications.
